# Force generation and resistance in human mitosis

**DOI:** 10.1007/s12551-024-01235-0

**Published:** 2024-09-28

**Authors:** Colleen C. Caldwell, Tinka V. M. Clement, Gijs J. L. Wuite

**Affiliations:** Department of Physics and Astronomy, and LaserLaB Amsterdam, Vrije Universiteit Amsterdam, De Boelelaan 1081, 1081 HV Amsterdam, The Netherlands

**Keywords:** Mitosis, Chromosome, Spindle, Centromere

## Abstract

Since the first observations of chromosome segregation over 150 years ago, efforts to observe the forces that drive mitosis have evolved alongside advances in microscopy. The mitotic spindle acts as the major generator of force through the highly regulated polymerization and depolymerization of microtubules as well as associated motor proteins. Centromeric chromatin, along with associated proteins including cohesin and condensin, is organized to resist these forces and ensure accurate chromosome segregation. Microtubules and centromeric chromatin join at the kinetochore, a complex protein superstructure. Ongoing research into the forces generated at the kinetochore-microtubule interface has resulted in a range of estimates for forces necessary to separate chromosomes, from tens to hundreds of piconewtons. Still, the exact magnitude and regulation of these forces remain areas of continuing investigation. Determining the precise forces involved in chromosome segregation is hindered by limitations of current measurement techniques, but advances such as optical tweezers combined with fluorescence microscopy are promising for future research.

## Introduction

Observations of segregation of chromosomes during mitosis have been ongoing for 150 years, developing alongside advances in microscopy and other technologies (Schneider 1873; Strasburger 1876; McIntosh and Hays 2016). In this visually striking cellular process, replicated chromosomes are compacted into masses so dense they can be observed by light microscopy, and subsequently pulled apart. This pulling apart of chromosomes has long been speculated to involve substantial cellular forces (McIntosh and Hays 2016). However, what exactly the role, source and magnitude of involved forces is has taken many decades to elucidate and is still not fully understood. Here, we give a concise overview on how forces are generated and resisted in the context of mitosis in human cells, with a focus on the process of chromosome segregation.

## Mitotic spindle

**Fig. 1:**
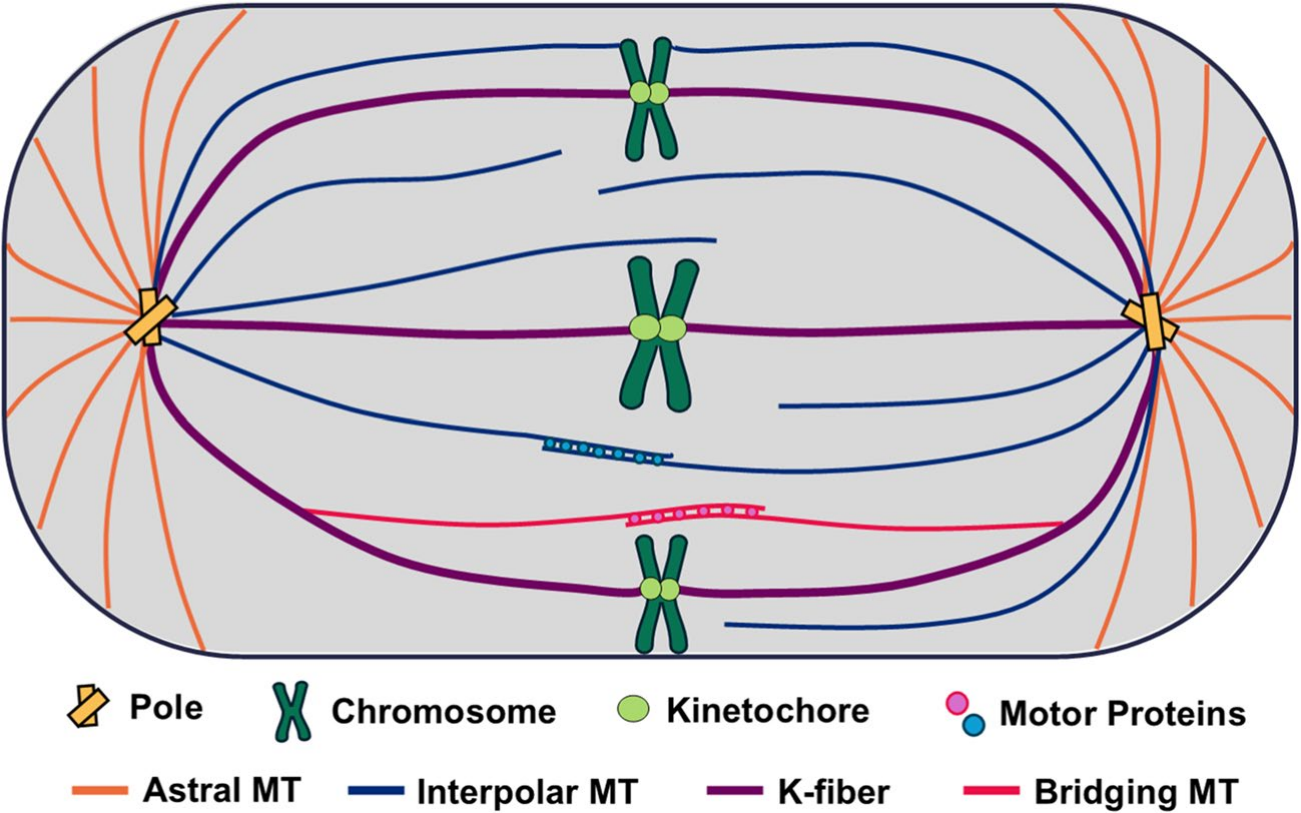
Schematic illustrating the major structural components of the mitotic spindle

The mitotic spindle is the cytoskeletal structure that generates the forces necessary to drive the separation of sister chromatids during cell division. The machinery of the mitotic spindle can be broken down into several primary components (Fig. 1). At each pole is a centrosome, an organelle containing two centrioles, made up of microtubules (MTs), and surrounded by a dense protein structure. Leading up to mitosis, the centrosomes increase the rate of MT nucleation in order to form the spindle poles (Vasquez-Limeta and Loncarek 2021). In metaphase, the chromosomes are aligned at the centre of the cell, called the metaphase plate. Each sister chromatid possesses a kinetochore, a protein structure located at the centromere to which MTs may attach. The majority of the spindle is composed of microtubules, the distinct types of which will be discussed.

**Fig. 2:**
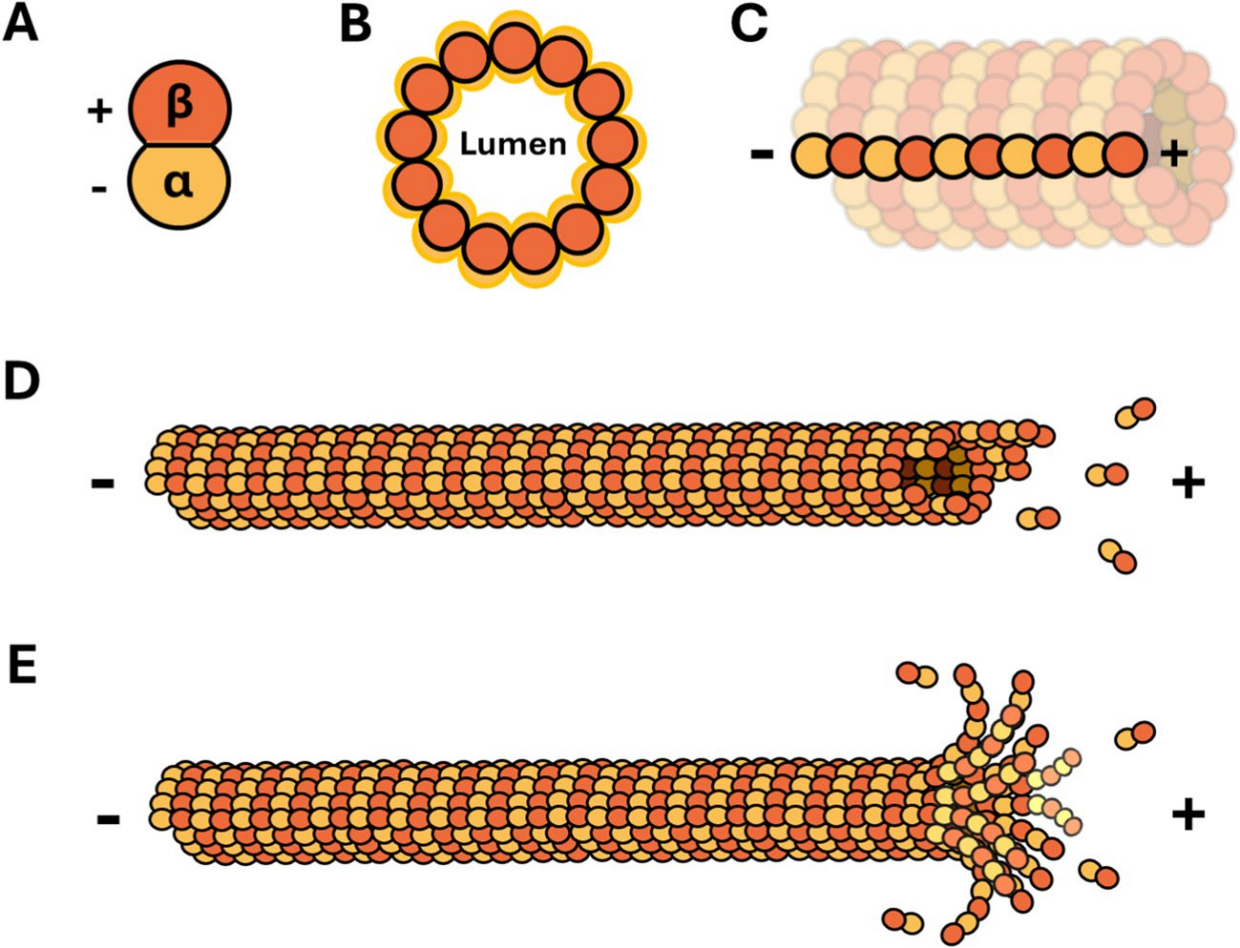
Structural basis of microtubules illustrating **A** the tubulin dimer, **B** cylindrical structure of protofilaments, **C** protofilaments forming a microtubule, and **D/E** microtubule polymerization/depolymerization

Microtubules are composed of polymers of α and β tubulin. A heterodimer is first formed between α and β tubulin monomers, which is the subunit that will assemble during polymerization (Nogales et al. 1998). The end at which the α tubulin monomer is exposed is referred to as the minus (-) end while the end at which the β tubulin monomer is exposed is referred to as the plus ( +) end (Fig. 2a). Tubulin protofilaments are formed by the end-to-end association of heterodimers, with 13 protofilaments assembling to form the microtubule (Fig. 2c). The hollow space within the cylindrical structure is the lumen (Fig. 2b). During polymerization, the rate of addition is much greater at the plus end of the microtubule (Allen and Borisy 1974). Microtubule polymerization/depolymerization (Fig. 2d,e) is a major generator of both pushing and pulling forces driving chromosome segregation and is a target of tight regulation by a range of factors (Gudimchuk and McIntosh 2021). The underlying mechanism of forces generated by polymerization/depolymerization can be thoroughly understood as a thermal ratchet (Dogterom et al. 2002).

## Microtubules of the mitotic spindle

### Astral microtubules

Astral microtubules (aMT) play a critical role in establishing the positioning the spindle poles during mitosis. They orient with their minus ends toward the poles and their plus ends radiating toward the cell cortex. While the primary role of the aMTs is positioning the spindle apparatus and determining the orientation of cell division, they also play a role in balancing the forces of the spindle and maintaining spindle stability (Bouvrais et al. 2021).

### Interpolar microtubules

Interpolar microtubules (iMT) are oriented with their minus ends toward the spindle pole and their plus ends overlapping in the central plane of the cell in a midzone microtubule bundle (Lee et al. 2012). They play critical roles in the maintenance of the elongated spindle structure as well as chromosome segregation. Another subset of MTs at the central plane are bridging microtubules (bMTs), which also possess overlapping plus ends at the midzone, but have minus ends that are localized to kinetochore microtubules (kMTs) (Vukušić et al. 2017). bMTs drive the curving of linked K-fibers and withstand tension to maintain the tensile forces between sister kinetochores (Kajtez et al. 2016). The crosslinked bMTs provide reinforcement in the region surrounding the chromosomes that allows perturbations to be resisted (Suresh et al. 2020).

### Kinetochore microtubules

Like interpolar microtubules, kMTs orient with their minus end toward the pole and their plus end toward the chromosomes at the central plane of the cell. The plus end of the kMT associates with the kinetochore. Each kinetochore associates with a quantity of kinetochore microtubules, the number of which varies across species. The fiber formed at each kinetochore is termed the K-fiber. In human cells approximately 10–20 kMTs form the K-fiber, though only half extend from the kinetochore to the spindle pole while the rest terminate prior to reaching the pole (Wendell et al. 1993; O’Toole et al. 2020; Kiewisz et al. 2022).

In addition to the MTs, an essential component of the K-fiber that allows force generation is the kinetochore. The kinetochore is a macromolecular protein structure that assembles at the centromere of each sister chromatid. The inner kinetochore is associated with the chromatin at the centromere while the outer kinetochore mediates interactions with the microtubules. The constitutive centromere-associated network (CCAN) assembles at the inner kinetochore and also recruits components of the outer kinetochore that will drive kMT attachment (Cheeseman and Desai 2008). The KMN network that mediates kMT attachment is composed of three complexes: KNL1, MIS12, and NDC80 (Yatskevich et al. 2024). Direct contacts with the plus end of the kMTs are made by NDC80 and KNL1. Multiple complexes interacting with each kMT establish stable interactions that can withstand the forces generated during chromosome segregation (Joglekar et al. 2006; Cheeseman et al. 2006). Other kinetochore components play a role in regulating the polymerization state of the MT. These and motor proteins associated with kMTs will be discussed below.

## Motor proteins

The forces necessary for the generation and maintenance of the mitotic spindle and separation of chromosomes are also reliant on motor proteins (Titus and Wadsworth 2012). This class of proteins binds to microtubules and generates force through the hydrolysis of ATP. They may be separated into the classes of kinesins and dyneins.

### Kinesins

The kinesin superfamily is divided into 14 families, all of which are characterized by a motor domain which binds MTs and usually drives directional movement as ATP is hydrolyzed (Hunter and Allingham 2020). Kinesin-5 family proteins are particularly important during mitosis. They form homotetramers that possess two linked motor domains and are able to crosslink anti-parallel iMTs (Sharp et al. 1999; Wildenberg et al. 2008). While each motor domain moves towards the plus end of the linked MTs they generate anti-parallel sliding (Kapitein et al. 2005). Some kinesin-5 proteins also localize to the ends of MTs and regulate depolymerization rates (Pandey et al. 2021). Kinesin-12 has a similar and partially redundant function with kinesin-5 proteins (Drechsler and McAinsh 2016).

Kinesin-13 proteins are another important set of motor proteins in chromosome segregation, playing a crucial role in the regulation of microtubule depolymerization state. These proteins localize to K-fibers both at the pole and central plane, where they maintain stable length during metaphase (Barisic and Rajendraprasad 2021). They also localize to spindle microtubules at the central plane and kinetochores where they promote rapid depolymerization of kMTs during chromosome segregation (Manning et al. 2007). Instead of hydrolyzing ATP in order to translocate along MTs, they catalyze release of tubulin dimers (Desai et al. 1999; Trofimova et al. 2018). Members of the kinesin-4 family also similarly promote microtubule disassembly (Akhmanova and Kapitein 2022). While the yeast kinesin-8 family protein clearly promotes microtubule disassembly, human kinesin-8 protein Kif18A may instead suppress dynamics as a plus end capping factor (Shrestha et al. 2018). Kinesin-8, 4, and 10 family proteins work together to promote stable centering of kinetochores. Kinesin-8 inhibits kinetochore movement by suppressing K-fiber dynamics in a length-dependent manner, while kinesin-4 and 10 oppose each other to modulate kinetochore tension and polar ejection forces (Stumpff et al. 2012). Other families of kinesins play roles during mitosis but not in chromosome segregation. The roles of each kinesin family in the establishment of the mitotic spindle structure have been discussed in previous reviews (Titus and Wadsworth 2012; Ali and Yang 2020).

### Dynein

While there are a range of dyneins in human cells, the most relevant to chromosome segregation in mitosis is cytoplasmic dynein 1 (Hinchcliffe and Vaughan 2018). This large protein (> 1MDa) is composed primarily of a homodimer that makes up the motor domain which hydrolyses ATP to drive the stepping motion towards the plus end of MTs (Reck-Peterson et al. 2006). Dynein plays roles throughout mitosis, partially regulated by its spatial localization at kinetochores, MTs, or the cellular cortex (Raaijmakers and Medema 2014). Dynein is recruited to the outer kinetochore through interactions between its partner, dynactin, Spindly, and kinetochore associated complexes including Mis12 and KLN1 (Kops et al. 2005; Sacristan et al. 2018). At the kinetochore, dynein strips and carries away kinetochore components as cargo, stabilizing the kMT attachment and silencing the checkpoint so mitosis may proceed (Howell et al. 2001; Auckland et al. 2020). While roles for kinetochore dynein have been suggested in chromosome alignment and anaphase separation, the exact function is unclear (Raaijmakers and Medema 2014). On iMTs, dynein acts in opposition to the kinesin-5 protein, applying force to generate anti-parallel sliding of adjacent iMTs in opposite directions (Ferenz et al. 2009). At the cellular cortex, dynein is recruited to nuclear mitotic apparatus (NuMA), where dynein generates force via interaction with associated aMT (Okumura et al. 2018).

To complicate the generation of forces by both kinesin and dynein motor proteins described above, the generation of rotational forces has also been observed. Kinesin-14, 5, and 8 have all be observed to drive rotation of microtubule in vitro, producing enough torque to result in the twisting during anti-parallel microtubule sliding (Yajima et al. 2008; Mitra et al. 2018, 2020). Cytoplasmic dynein has also been observed in vitro to follow a helical path, with a preference for right-handed helical motion which may provide a mechanism to avoid roadblocks (Can et al. 2014). The shape adopted by the spindle suggests the presence of rotational forces, though further study is required to better understand the mechanisms of generation and significance (Tolić et al. 2019).

In addition to the active force generation of the motor proteins, passive crosslinking proteins play a role in the response to force. At the spindle midzone anaphase spindle elongation 1/protein regulator of cytokinesis 1/microtubule associated protein 65 (Ase1/PRC1/MAP65) family proteins crosslink microtubules and serve as a regulatory platform in spindle assembly and maintenance (She et al. 2019). Though they are non-motor proteins, AseI generates several pN forces due to entropic expansion as it diffuses which can oppose microtubule sliding by motor proteins (Lansky et al. 2015). NuMA is a multi-functional protein critical for spindle structure that localizes to the poles and cortex recruiting dynein and dynactin at the minus end of aMTs (Kiyomitsu and Boerner 2021). The interplay of active and passive force generators on the spindle has been previously reviewed (Elting et al. 2018).

## Mechanisms of force generation

With the basic components of the mitotic spindle illustrated, we may discuss how and where they work together to generate the forces that align and then segregate chromosomes during mitosis. Before chromosome segregation, chromosomes are first aligned at the metaphase plate. This striking movement of chromosomes was an early visual clue to the forces that are involved. Several models focusing on the forces driving chromosome alignment have been previously reviewed (Risteski et al. 2021).

### Kinetochore-MT interface

During mitosis, a critical site of force generation occurs at the kMTs and kinetochore. Once kMTs from opposite poles associate with the kinetochores at each sister chromatid during prometaphase, they apply equivalent pulling forces, resulting in relatively static chromosomes (McNeill and Berns 1981; Hays and Salmon 1990). Increased tensions between 1–5 pN stabilize the budding yeast kMT-kinetochore attachment (Akiyoshi et al. 2010). While the chromosome appear relatively static, they exhibit oscillations that reduce incorrect kinetochore-microtubule associations (Iemura et al. 2021). Interplay between proteins that promote oscillations, such as kinesin-13, and others that suppress oscillations, such as kinesin-8 and CLASPs, regulate kinetochore dynamics (Risteski et al. 2021). Oscillation periods also were modulated by the stiffness of the linkage between sister kinetochores (Jaqaman et al. 2010). Following alignment and checkpoint verification for proper orientation, anaphase begins (McAinsh and Kops 2023). The cohesin linking sister chromatids is cleaved and they are pulled towards opposite spindle poles (Hauf et al. 2001).

While motor proteins are essential at the kinetochore, they do not appear to be primary generators of force there due to their motile activity, but instead maintain the connection with the depolymerizing K-fiber (Raaijmakers and Medema 2014; Ali and Yang 2020). The depolymerization of the kMTs of the K-fiber applies poleward force to the chromosomes while Ndc80 kinetochore associated proteins maintain attachment to the shrinking fiber (Powers et al. 2009). A single depolymerizing kMT in budding yeast results in a force up to 11 pN (Akiyoshi et al. 2010). Human K-fibers contain 10–20 kMTs, suggesting the potential to generate higher forces (Wendell et al. 1993; O’Toole et al. 2020; Kiewisz et al. 2022).

### Interpolar and astral microtubules

In addition to the forces kMTs apply to chromosomes are opposing forces from iMTs and aMTs that drive chromosomes away from the poles, termed polar ejection forces (PEF) (Ault et al. 1991; Rieder and Salmon 1994). Mitotic spindle positioning requires dynamic interplay between aMTs and the Gα_i_–LGN–NuMA–dynein complex (Lechler and Mapelli 2021). Chromokinesins, particularly of kinesin-4 and 10 families, interact with both iMTs and chromosomes and are critical to the generation of PEFs (Wandke et al. 2012; Almeida and Maiato 2018). Kid, a family 10 kinesin, drives PEF while Kif4A, a family 4 kinesin, antagonizes PEF by supressing microtubule polymerization (Stumpff et al. 2012). Other iMT associated forces occur without any interaction at chromosomes. Anti-parallel sliding of iMTs is driven by kinesin-5 family motor proteins that link iMT from opposite poles (Kapitein et al. 2005). These same kinesin-5 proteins also regulate force generation by altering the depolymerization rate of iMTs (Pandey et al. 2021). While PEFs are likely substantially lower than the forces generated at kinetochores, they are essential in establishing kinetochore-MT attachments (Drpic et al. 2015). Another driver of chromosome centering has been identified in the poleward flux, where the minus end depolymerizes as the plus end polymerizes (Mitchison 1989). As a chromosome departs from the center, the longer K-fiber increases poleward flux to drive the chromosome back to the center. The coupling of bMTs and kMTs by NuMA allows bMTs undergoing flux to transmit forces to K-fibers, increasing their rate of flux and driving chromosome centering (Risteski et al. 2022).

### Methods of force detection

A range of strategies to detect force has resulted in a wide range of observed forces in the separation of chromosomes. Early estimates were low (~ 0.1 pN) and assumed that the forces involved were only required to overcome the fluid drag force to generate similar velocity between chromosomes of different sizes in the viscous cytoplasm (Nicklas 1965). Two decades later, Nicklas used microneedles in grasshopper spermatocytes to oppose chromosome separation during anaphase and observed that stall forces as high as ~ 700 pN may be required to halt motion (Nicklas 1983).

In vitro assays have been used to probe the forces generated by individual components of the mitotic spindle. Optically trapped beads coated with kinetochore particles were used to apply tension to surface tethered microtubules and observe stabilization induced by increased tension with rupture forces of ~ 9 pN (Akiyoshi et al. 2010). Similarly, MTs on the surface may be crosslinked to another MT by motor or non-motor proteins, while force production is measured via an optically trapped bead tethered to the crosslinked MT (Palumbo et al. 2022). This revealed that a single kinesin-5 molecule exerts approximately 1.5 pN of force to induce antiparallel sliding of MTs (Shimamoto et al. 2015). PEFs have been approximated in vitro by adhering chromosomes to surface and reconstituting MTs on a bead trapped in an optical tweezer, measuring forces of 1–3 pN (Brouhard and Hunt 2005).

In order to probe forces within living cells, fluorophores have been introduced to components of the kinetochore that allow for distance changes induced by force to be interpreted through changes in Förster Resonance Energy Transfer (FRET) efficiency. Salmon and Bloom reviewed a range of FRET tension sensors that were used to estimate the tension force at the kinetochore (Salmon and Bloom 2017). Introducing a FRET pair into *Drosophila* CENP-C, forces of 1–2 pN per molecule were measured and used to estimate a total force of 144–764 pN at each kinetochore (Ye et al. 2016). Similar assays introducing FRET sensors into NDC80 of budding yeast estimated forces of ~ 2–6 pN per kMT during mitosis (Suzuki et al. 2016). Together, these experiments illustrate the wide range of observed and calculated spindle forces. Though these literature values can be difficult to reconcile, we speculate that the force necessary for mitotic progression in unperturbed human cells is likely at least tens to several hundred piconewtons.

## Structure of human centromeres

### Resisting force

Given the force-generating capacity of the mitotic spindle, the compliant centromeric chromatin must be organized in a way that protects it from significant force-induced deformation. Although centromeric chromatin stretches in response to spindle forces, it is much more rigid than “bare” chromatin (Bloom 2008; Harasymiw et al. 2019). To understand how the centromere achieves these mechanical properties, it is helpful to start with a description of centromeric chromatin.

### Centromeric chromatin

**Fig. 3:**
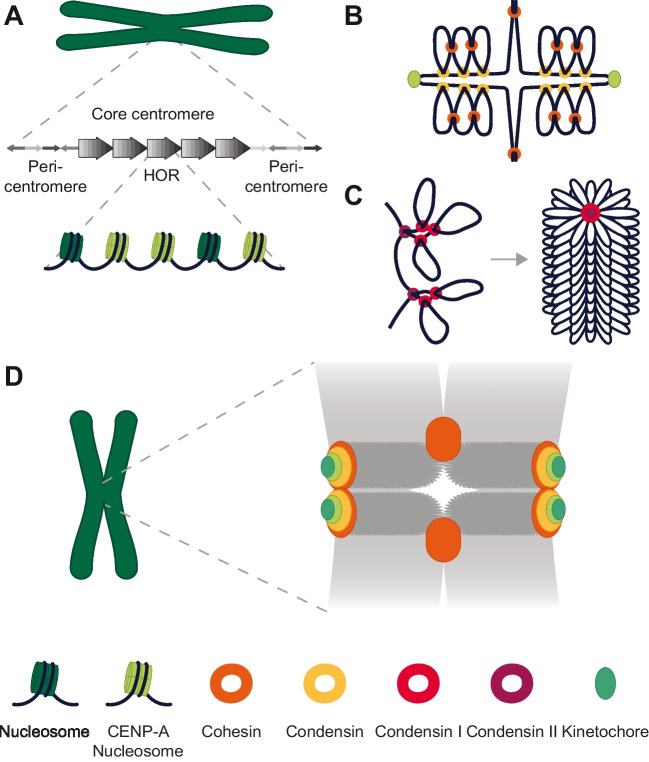
Composition and organization of human centromeres. **A** Schematic depiction of a human centromere (Aldrup-MacDonald and Sullivan 2014; McKinley and Cheeseman 2016). **B** Schematic depiction of yeast centromere (Lawrimore and Bloom 2022). **C** Formation of bottlebrush structure of human chromosomes (Gibcus et al. 2018). **D** Model of human centromere organization (Sacristan et al. 2024)

Humans have regional centromeres consisting of core centromeres flanked by pericentromeres. The core centromere is mainly composed of arrays of 171 bp α-satellite DNA, formed into higher order repeats (HORs) that are themselves reiterated thousands of times to span up to 5 Mbp (Manuelidis 1978; Aldrup-MacDonald and Sullivan 2014; Altemose et al. 2022a). A subset of nucleosomes found on HORs contain the histone H3 variant CENP-A (Fig. 3a) (Allshire and Karpen 2008; Bodor et al. 2014; McKinley and Cheeseman 2016). In contrast, pericentromeres contain divergent arrays that lack higher order repeats and are devoid of CENP-A (Fig. 3a) (Aldrup-MacDonald and Sullivan 2014; Altemose et al. 2022b).

### Kinetochores

Two CCAN proteins, CENP-C and CENP-N, specifically recognize CENP-A-containing nucleosomes (Carroll et al. 2010). Along with the action of other proteins, this recognition results in kinetochore assembly at a CENP-A rich region of the core centromere, facilitating attachment and anchoring of spindle microtubules to centromeric chromatin (Cheeseman and Desai 2008; McKinley and Cheeseman 2016). In addition to force transmission, the kinetochore is responsible for the spindle assembly checkpoint (SAC), which prevents chromosome segregation before all chromosomes have been properly attached to kMTs (Musacchio and Salmon 2007).

### Centromere-associated proteins

Several proteins play an important role in organizing centromeric chromatin, in turn enabling it to resist mechanical forces that are imposed by the mitotic spindle and transduced by the kinetochore. Most important are the SMC-family proteins cohesin and condensin. Cohesin and condensin are ring-shaped proteins that can entrap two strands of DNA in their lumen (Hirano 2006). Condensin is thought to form loops within each chromatid, offering the centromeric chromatin the rigidity it requires to resist spindle forces (Hirano 2006; Ribeiro et al. 2009). Cohesin can simultaneously entrap a stand of DNA from each sister chromatid, leaving them interlinked and unable to separate prematurely (Michaelis et al. 1997; Uhlmann et al. 1999; Haering et al. 2008). Early in mitosis, cohesin is present along the entire length of the chromosome. During prophase, cohesin along the chromosome arms is removed by WAPL, whereas cohesin in centromeric regions is protected from WAPL-mediated degradation by Shugoshin (Waizenegger et al. 2000; McGuinness et al. 2005; Gandhi et al. 2006). This centromeric cohesin pool is finally cleaved by separase when the SAC has been satisfied at the onset of anaphase, allowing sister chromatids to move towards opposite spindle poles (Hauf et al. 2001; Musacchio and Salmon 2007).

### Higher-order organization of centromeric chromatin

Much of the knowledge on the organization of centromeres has been gained by study of budding yeast. Budding yeast contains a point centromere to which a single microtubule attaches, flanked by pericentromeric regions (Yeh et al. 2008; Lawrimore and Bloom 2019). The simplicity of this centromere made it a valuable starting point for the study of centromere organization. In budding yeast, the pericentromere forms a radial loop or bottlebrush structure. In this structure, condensin sits at the base of the loops, and cohesin is located at the loop periphery and acts as a crosslinker (Fig. 3b) (Stephens et al. 2013; Lawrimore et al. 2018; Lawrimore and Bloom 2022). 3D simulations of the budding yeast centromere highlighted the different contributions of cohesin and condensin to the observed tension landscape (Lawrimore et al. 2016). In unperturbed cells, tension is predicted to be higher in axial chromatin, where the condensin molecules are located, than in the radial loops. In the absence of cohesin, the tension landscape will look similar. In absence of condensin, however, tension is amplified in cohesin rings, as in this model they are now the only remaining factors crosslinking the centromeric chromatin. Finally when both cohesin and condensin are absent, tension is evenly distributed over the pericentromeric chromatin (Lawrimore et al. 2018). Together, these lines of evidence elucidate how the cross-linked loop structure gives the (peri)centromere the rigidity required to withstand pulling forces from the spindle (Bouck and Bloom 2007; Stephens et al. 2011; Lawrimore and Bloom 2019).

In human cells, mitotic chromatin is similarly organized into a bottlebrush-structure, with condensin II forming loops at the base of the bottlebrush, and condensin I creating sub-loops (Fig. 3c) (Gibcus et al. 2018). The organization of the centromere is less well defined. It was recently found that centromeric DNA forms compact structures, such as hairpins, while the centromere-associated protein CENP-B further aids in its compaction (Chardon et al. 2022). Furthermore, Sacristan et al. recently found compelling evidence that human centromeres are bipartite: each chromatid has two separate centromere subdomains, on which two separate kinetochores form, which can attach to kMTs independently (Sacristan et al. 2024). CENP-A within chromatids was similarly observed to be stretched and/or appearing in several clusters in human cells in two other recent studies, although they did not interpret this as bipartite (Di Tommaso et al. 2023; Sen Gupta et al. 2023). Combining insights from these three recent studies allows us to come up with an updated model of human centromere organization (Fig. 3d). Centromeric α-satellite DNA, as well as likely pericentromeric DNA, is present between the CENP-A signal of both sister chromatids. CENP-A concentrates in clusters along the contour of α-satellite DNA near the outer surface of the chromatids (Di Tommaso et al. 2023; Sen Gupta et al. 2023; Sacristan et al. 2024). Kinetochores form on top of each of the two CENP-A clusters, and can independently form attachments to kMTs (Sacristan et al. 2024). Cohesin is enriched in pericentromeric regions and relatively depleted in core centromeres (Sen Gupta et al. 2023). The largest pool of cohesin lines the interface between sister chromatids near the centromeres, but shows a significant dip in the centre, indicating a relatively depleted zone (Sen Gupta et al. 2023; Sacristan et al. 2024). This pool likely reflects the cohesin that tethers the sister chromatids. A second smaller pool of cohesin is present proximal to the CENP-A signal (Sen Gupta et al. 2023; Sacristan et al. 2024). Reducing the pools of centromeric cohesin showed that the CENP-A-proximal cohesin likely functions to tether the centromere subdomains and prevent incorrect (merotelic) attachments to kMTs (Sacristan et al. 2024). Condensin, especially condensin II, is enriched at centromeres compared to the rest of the chromosome (Sacristan et al. 2024). It is located near CENP-A, mostly overlapping with cohesin. In condensin-depleted cells, bipartite centromere organization is abolished, indicating that condensin plays an important role in establishing bipartition (Sacristan et al. 2024). Each centromere subdomain is closely associated with the adjacent pericentromeric region from the same arm. Interestingly, modelling indicated that a bottlebrush-like organization of pericentromeres could promote the partitioning of the core centromere into two subdomains (Sacristan et al. 2024), perhaps linking the newly found organization of human centromeres to the model proposed for budding yeast centromeres. Future studies are required to investigate how bipartite centromere organization affects kMT attachment, error correction, and potentially force generation.

### Force throughout mitosis

Although the mitotic spindle could likely produce forces up to many hundreds of piconewtons, it likely does not produce a constant force throughout mitotic progression. Force has been argued to be highest during prometaphase as chromosomes are actively aligned. Here, spindle forces are likely also an important factor in regulating mitotic progression (Asbury 2017). Spindle forces are thought to be lowest during anaphase, when remaining connections between sister chromatids are severed. Traditionally, it was thought that in this mitotic phase only viscous drag of the surrounding cytoplasm needs to be overcome in order to move chromatids polewards (Nicklas 1965). However, remaining DNA intertwines or ultrafine bridges (UFBs) between sister chromatids are known to persist into anaphase even in unperturbed cells. Spindle forces are required to resolve these UFBs and prevent segregation errors (Baumann et al. 2007; Spakman et al. 2022). Therefore, calculations of anaphase spindle forces that rely only on overcoming viscous drag of the cytoplasm are likely underestimates of the true forces. Taken together, spindle forces play an active role in regulating mitotic progression, from prometaphase all the way into anaphase.

## Conclusions

In this review, we have described current literature on how forces in human mitosis are generated by the spindle and resisted by the centromere (see Table 1 for an overview of discussed proteins and their functions). The forces that drive chromosome segregation are mainly generated by depolymerization of kMTs. Force estimates are usually experimentally obtained via relatively crude methods or in vitro reconstitution of individual components and vary widely. In human centromeres, a complex network of proteins cooperates to give the centromeric chromatin the flexibility and rigidity required to respond to spindle forces. Aside from physically separating sister chromatids, spindle forces play a regulatory role, from prometaphase all the way into anaphase. A current limitation in the field of mitotic force appears to be the lack of precise and direct measurement of spindle forces that are applied during normal mitosis, minimum forces required to achieve successful mitosis, and material properties of centromeres. With recent advancements of accurate single-molecule techniques, perhaps these points could be elucidated in the near future. For example, optical tweezers combined with fluorescence microscopy have recently been used to apply forces to whole mitotic chromosomes and extract their mechanical properties (Meijering et al. 2022). As this technique is capable of exerting forces in the range we expect to occur in mitosis, it could provide an interesting avenue for the study of forces involved in human mitosis and chromosome segregation in particular.

**Table 1:** Overview of discussed key proteins

Category	Sub-category	Protein (family/complex)	Key function(s)
Spindle microtubules	-	α tubulin	Building block of spindle microtubules
		β tubulin	Building block of spindle microtubules
Kinetochore	Inner kinetochore	CCAN (including CENP-C, CENP-N)	Kinetochore assembly, recognizes CENP-A
	Outer kinetochore	KMN network (including NL1, MIS12, and NDC80)	Mediates kMT attachment
Motor proteins	Kinesins	Kinesin-4 family (including Kif4A)	Promotes microtubule disassembly, opposes kinesin-10 to modulate kinetochore tension, interacts with iMTs, antagonizes PEFs
		Kinesin-5 family	Generates anti-parallel sliding forces, regulate MT depolymerization rates, opposes dynein on iMTs, drives microtubule rotation
		Kinesin-8 family (including Kif18A)	Supresses k-fiber dynamics (in length-dependent manner), drives microtubule rotation
		Kinesin-10 family (inlcuding Kid)	Opposes kinesin-4 to modulate kinetochore tension, drives PEFs
		Kinesin-12 family	Function similar to kinesin-5 family
		Kinesin-13 family	Regulate microtubule depolymerization state
		Kinesin-14 family	Drives microtubule rotation
	‒	Dynein	Drives stepping motion towards + end of MTs, carries kinetochore components as cargo, stabilizes kMT attachments, opposes kinesin-5 on iMTs to generate antiparallel sliding
Non-motor microtubule associated proteins	‒	Ase1/PRC1/MAP65 family	Crosslink microtubules, serves as regulatory platform
		Gαi–LGN–NuMA	Forms complex with dynein to position the spindle
Centromeric chromatin	‒	CENP-A	Signals location of kinetochore assembly
Centromere-associated proteins	SMC-family	Cohesin	Links sister chromatids, crosslinks chromatin loops
		Condensin	Organizes chromatin by loop formation
	‒	WAPL	Removes cohesin from chromosome arms
		Shogoshin	Protects centromeric cohesin from WAPL-mediated degradation
		Separase	Cleaves centromeric cohesin
		CENP-B	Aids in compaction of centromeric chromatin

## Data Availability

No datasets were generated or analysed during the current study.
